# Importance of cardiac-synchronized vagus nerve stimulation parameters on the provoked chronotropic response for different levels of cardiac innervation

**DOI:** 10.3389/fphys.2024.1379936

**Published:** 2024-05-21

**Authors:** Max Haberbusch, Bettina Kronsteiner, Philipp Aigner, Attila Kiss, Bruno Karl Podesser, Francesco Moscato

**Affiliations:** ^1^ Center for Medical Physics and Biomedical Engineering, Medical University of Vienna, Vienna, Austria; ^2^ Ludwig Boltzmann Institute for Cardiovascular Research, Vienna, Austria; ^3^ Center for Biomedical Research and Translational Surgery, Medical University of Vienna, Vienna, Austria; ^4^ Department of Cardiac Surgery, Medical University of Vienna, Vienna, Austria; ^5^ Austrian Cluster for Tissue Regeneration, Vienna, Austria

**Keywords:** vagus nerve stimulation, stimulation parameters, cardiac denervation, isolated heart, autonomic cardiac control

## Abstract

**Introduction:**

The influence of vagus nerve stimulation (VNS) parameters on provoked cardiac effects in different levels of cardiac innervation is not well understood yet. This study examines the effects of VNS on heart rate (HR) modulation across a spectrum of cardiac innervation states, providing data for the potential optimization of VNS in cardiac therapies.

**Materials and Methods:**

Utilizing previously published data from VNS experiments on six sheep with intact innervation, and data of additional experiments in five rabbits post bilateral rostral vagotomy, and four isolated rabbit hearts with additionally removed sympathetic influences, the study explored the impact of diverse VNS parameters on HR.

**Results:**

Significant differences in physiological threshold charges were identified across groups: 0.09 ± 0.06 μC for intact, 0.20 ± 0.04 μC for vagotomized, and 9.00 ± 0.75 μC for isolated hearts. Charge was a key determinant of HR reduction across all innervation states, with diminishing correlations from intact (*r* = 0.7) to isolated hearts (*r* = 0.44). An inverse relationship was observed for the number of pulses, with its influence growing in conditions of reduced innervation (intact *r* = 0.11, isolated *r* = 0.37). Frequency and stimulation delay showed minimal correlations (*r* < 0.17) in all conditions.

**Conclusion:**

Our study highlights for the first time that VNS parameters, including stimulation intensity, pulse width, and pulse number, crucially modulate heart rate across different cardiac innervation states. Intensity and pulse width significantly influence heart rate in innervated states, while pulse number is key in denervated states. Frequency and delay have less impact impact across all innervation states. These findings suggest the importance of customizing VNS therapy based on innervation status, offering insights for optimizing cardiac neuromodulation.

## 1 Introduction

Cardiac disorders stand as a formidable challenge to global health, driving the pursuit of innovative treatments that extend beyond pharmacological interventions (Ottaviani et al., 2022). Vagus nerve stimulation (VNS) emerges as a promising neuromodulation therapy, capitalizing on its capacity to finely tune heart rate and cardiac function. The therapeutic potential of VNS in cardiac applications hinges on a thorough comprehension of the interplay between the multifaceted vagus nerve which conveys sensory, motor, and visceral signals—and the complex cardiac innervation.

Unlike conventional pharmacological therapies, such as beta-blockers which broadly decrease heart rate and can impair the quality of life by restricting physical activity over extended periods, VNS offers a more targeted and controllable approach. The effects of VNS are immediate and cease when the stimulation is stopped, allowing patients, particularly those who are physically active, to better manage their heart rates in real-time. Furthermore, for patients with conditions like diabetes mellitus where beta-blockers are often contraindicated, VNS provides a vital alternative for managing persistent sinus tachycardia—a permanently elevated resting heart rate—thus broadening the scope of treatment options available and enhancing patient-specific care.

Advances in understanding of vagus nerve anatomy and VNS have illuminated its spatial effects (Settell et al., 2020; Kronsteiner et al., 2022; Blanz et al., 2023; Thompson et al., 2024); however, the temporal dimensions of its influence on the heart, particularly in different states of cardiac denervation, have remained under-investigated. Temporal parameters (e.g., stimulation frequency) and parameters influencing spatial distribution and number of activated nerve fibers (e.g., stimulation amplitude and pulse width), are thought to differentially affect heart rate modulation as shown in previous studies (Ojeda et al., 2016; Huffman et al., 2023). Nonetheless, the extent and nature of these effects, especially in conditions of different cardiac innervation, are not fully elucidated.

With these gaps in mind, this study aims to systematically analyze the influence of various cardiac-synchronized VNS parameters on heart rate modulation within different cardiac innervation conditions including intact innervation, bilateral vagal deafferentation, and complete vagal and sympathetic deafferentation. Our study adds to the understanding of how VNS parameters modulate heart rate responses in varied cardiac neural landscapes, thereby contributing to the development and refinement of VNS therapies for the treatment of cardiac diseases.

## 2 Materials and methods

**FIGURE 1:**
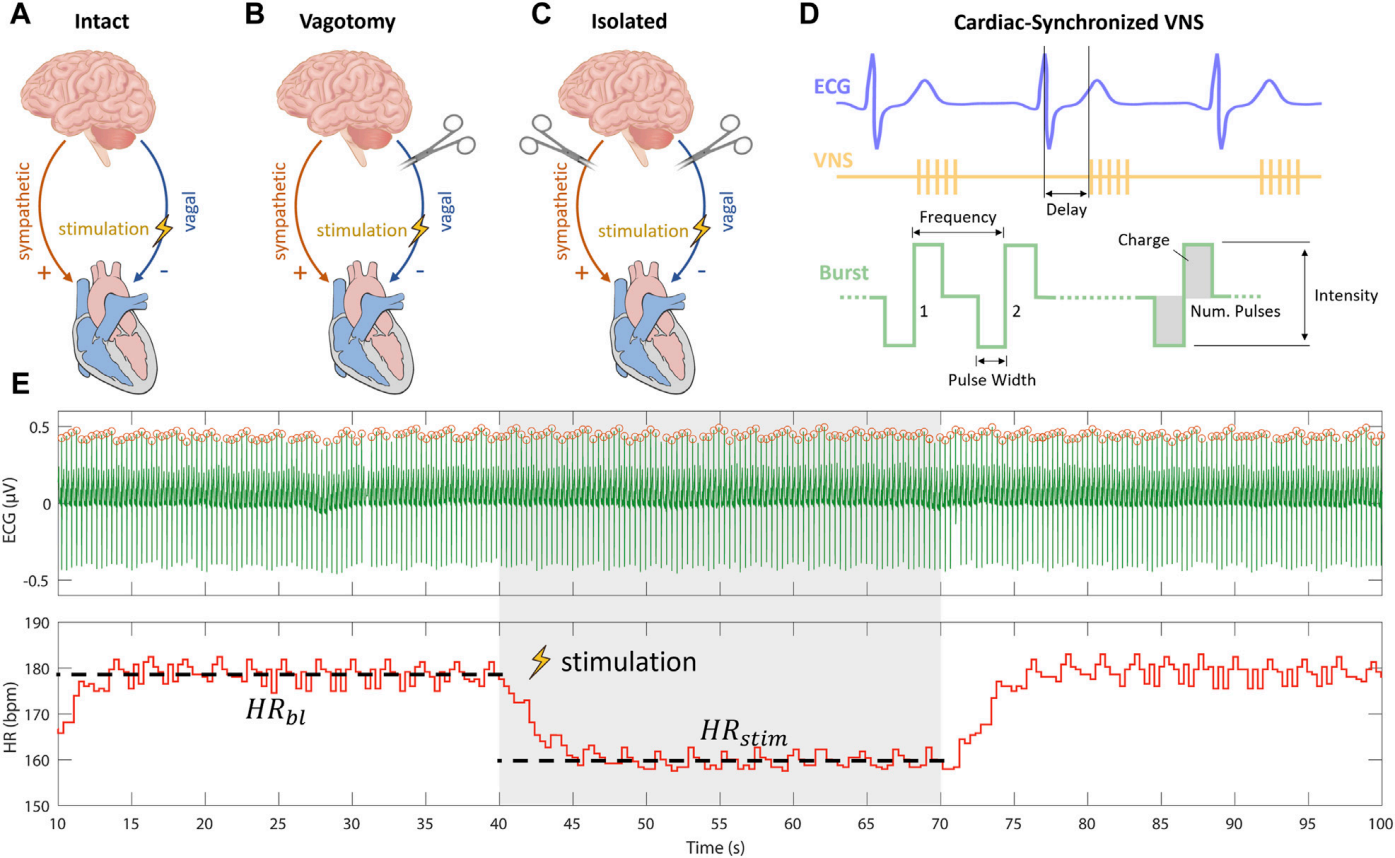
Experimental setups for vagus nerve stimulation (VNS) experiments including **(A)** intact cardiac innervation in sheep, **(B)** bilateral rostral vagotomy in rabbits, and **(C)** isolated rabbit hearts. **(D)** Schematic overview of cardiac-synchronized VNS with stimulation parameters. **(E)** Calculation of heart rate effect marker ( ΔHR ) from baseline ( ${HR}_{bl}$ ) and stimulation ( ${HR}_{stim}$ ) heart rates, extracted from a recording of VNS experiments.

To understand the varying impacts of cardiac-synchronized VNS parameters on heart rate under different cardiac innervation states, our study was structured following the protocol established by Ojeda et al. (2016). In brief, they used Latin Hypercube Sampling (LHS) to generate different combinations of VNS parameters to assess the variation in the provoked cardiac response including the influence on heart rate. Their research, focusing on sheep with intact cardiac innervation (Figure 1A), provided crucial insights into the acute cardiac effects of VNS. Notably, Ojeda et al. (2016) made their data publicly available, allowing for a comprehensive comparative analysis.

Building upon this foundation, our study extended the investigation to different innervation states. We first applied the approach used by Ojeda et al. (2016) on rabbits that underwent bilateral vagotomy rostral to the stimulation electrode (Figure 1B). This *in vivo* experimental setup, was used to isolate efferent vagal effects while maintaining the integrity of cardiac sympathetic pathways. Furthermore, to exclusively examine the efferent effects of VNS without sympathetic influences, we conducted *ex-vivo* experiments on isolated rabbit hearts (Figure 1C).

Our multi-layered experimental approach, was designed to analyze the influences of specific VNS parameters—such as stimulation intensity, pulse width, and frequency—on heart rate modulation across these varied cardiac innervation states. By comparing our findings from the rabbit models with the data from sheep experiments with intact cardiac innervation, as reported by Ojeda et al. (2016); (Kronsteiner et al., 2023), we aimed to provide a comprehensive understanding of how VNS parameters modulate heart rate under different cardiac innervation conditions.

### 2.1 Ethical compliance and animal subject details

All experimental protocols involving female New Zealand White rabbits (*n* = 5 for *in vivo* and *n* = 4 for *ex-vivo*, weighing 2.5–3.3 kg, aged 3–4 months) adhered to ethical standards and were approved by the Institutional Animal Care and Use Committee of the city of Vienna (Approval No. BMBWF 2020-0.016.858-GZ 2020-0.016.858). The procedures followed the ARRIVE guidelines, guaranteeing humane treatment and care of the animals under deep anesthesia and analgesia.

#### 2.1.1 Anesthesia and analgesia protocol

Anesthesia and analgesia protocols followed established procedures as detailed in our previous publication (Kronsteiner et al., 2023). In summary, premedication included ketamine (0.6 mL/kg) and dexmedetomidine (0.2 mL/kg). Anesthesia (sevoflurane 4%) was administered via an endotracheal tube, supplemented by continuous fentanyl infusion for analgesia. Fluid balance was maintained with crystalloid solution, and blood gases were monitored to ensure physiological conditions.

### 2.2 *In vivo* experiments

#### 2.2.1 Vagus nerve dissection and instrumentation

**FIGURE 2:**
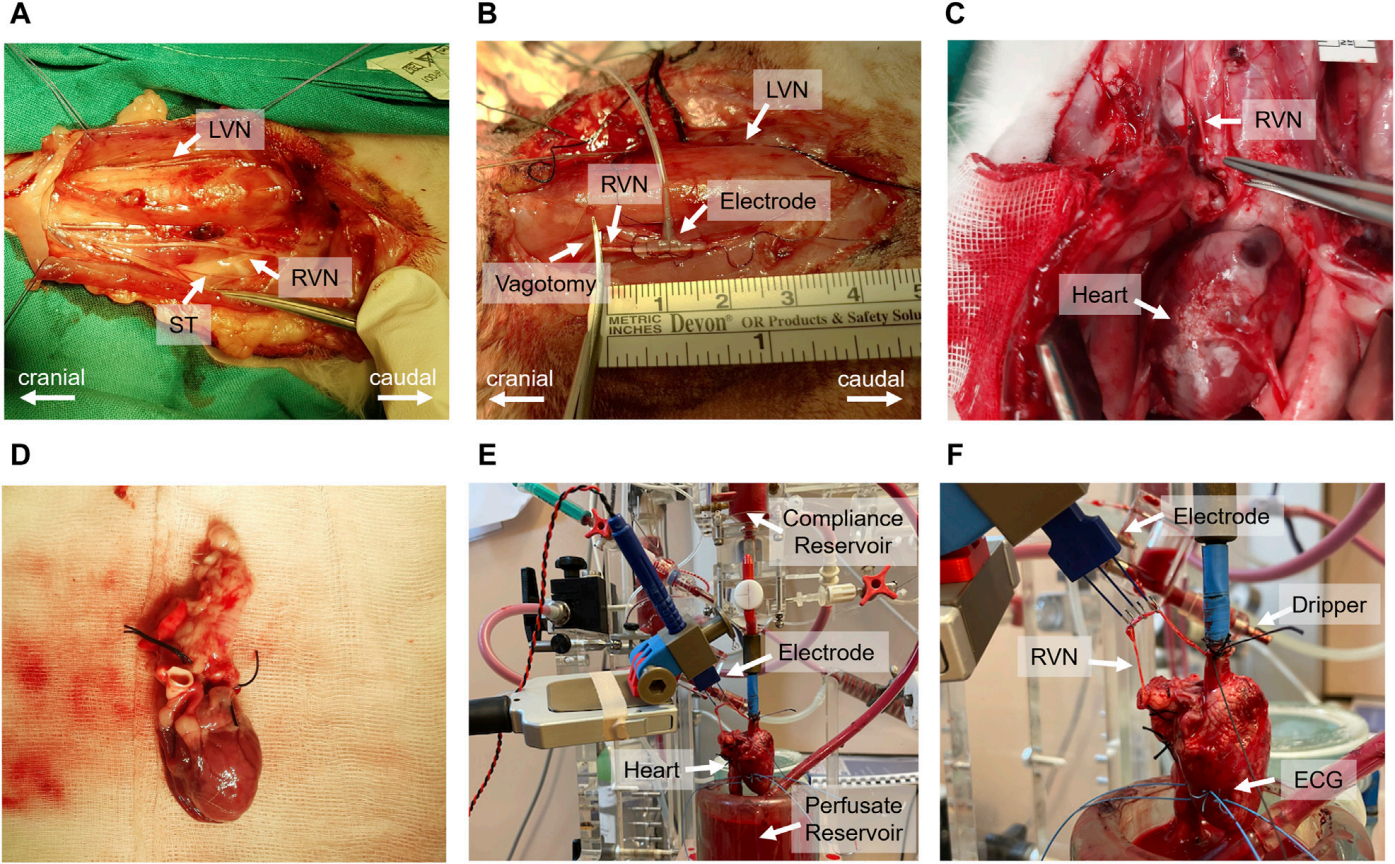
Comprehensive overview of the *in vivo* and *ex-vivo* stimulation experiments. **(A)** Surgical window highlighting the left vagus nerve (LVN), right vagus nerve (RVN) and sympathetic trunk (ST). **(B)** Electrode placement on the vagus nerve and the site of rostral vagotomy. **(C)** Sternotomy process and tracing of the vagus nerve to its entry into the epicardial fat pad. **(D)** Excised heart ready for immersion in ice-cold Krebs-Henseleit buffer. **(E)** Isolated heart system setup, including the compliance reservoir, hook electrode for stimulation, perfusate reservoir, and the isolated heart. **(F)** Close-up view of the isolated heart setup, indicating the positioning of the electrode on the right vagus nerve, the dripping system for vagus nerve moistening, and the wire electrodes implanted in the left ventricular wall for electrocardiogram (ECG) recording.

*In vivo* experiments followed the protocol outlined in Kronsteiner et al. (2023). Initially, a 3–5 cm surgical window was created at the cervical level to dissect the vagus nerve (Figure 2A). The carotid sheath was then carefully opened to expose the cervical vagus nerve which was isolated from surrounding tissues for application of a bipolar cuff electrode (Figure 2B). Following the electrode placement, a bilateral vagotomy was performed rostrally to the stimulation electrode. Standard 3-lead ECG recordings were acquired using needle electrodes placed on the chest and right hindlimb. At the completion of the experiments, the animals were humanely euthanized with pentobarbital.

### 2.3 *Ex-vivo* experiments

#### 2.3.1 Heart isolation

The *ex-vivo* experiments, including heart isolation, adhered to the protocol outlined in Kronsteiner et al. (2023). Initially, the vagus nerve was dissected from the cervical level to the superior cardiac branch. This was followed by a median sternotomy to access the thorax and pericardium, allowing the tracing of the right vagus nerve to its entry point into the epicardial fat pad (Figure 2C). Before severing the vagus nerve above the nodose ganglion, heparin was administered. Subsequently, the heart was excised (Figure 2D) and immediately placed in ice-cold Krebs-Henseleit buffer for preservation.

#### 2.3.2 *Ex-vivo* setup and instrumentation

Following dissection, the heart was cannulated and mounted onto an isolated heart system (Figure 2E) as outlined by Kronsteiner et al. (2023). Langendorff perfusion was maintained at a constant pressure of 80 mmHg using an erythrocyte-enriched Krebs-Henseleit buffer (Podesser et al., 1999). The vitality of the vagus nerve was preserved using a dripping system and the same buffer solution to ensure continuous moistness (Figure 2F). For stimulation, a bipolar triple hook electrode was positioned 4–5 cm above the superior cardiac branch. ECG measurements were conducted using two wire electrodes implanted in the left ventricular wall (Figure 2F).

### 2.4 Stimulation and data acquisition

Current-controlled stimulation was administered using an isolated linear stimulator. ECG measurements were captured by a differential amplifier, configured with a gain of 1,000, low-pass filtering at 1,000 Hz, and high-pass filtering at 1 Hz. Data were acquired and the stimulator was controlled using a real-time prototyping system, operating at a sampling rate of 1,000 Hz. We employed cardiac-synchronized stimulation, triggered by the R-peak in the ECG. The triggering of VNS was facilitated by a simple dynamic thresholding algorithm applied to the filtered ECG signal, as detailed in our previous work (Haberbusch et al., 2022). Cathodic-anodic charge-balanced rectangular pulses were employed. The adjustable parameters of stimulation included stimulation intensity in terms of current amplitude, frequency, pulse width, number of pulses, and onset delay of stimulation (Figure 1D).

#### 2.4.1 Stimulation protocol

Before commencing stimulation protocols, we established a stable baseline heart rate for at least 5 min in both *in vivo* and isolated heart experiments to ensure that any observed changes in heart rate were directly attributable to the stimulation rather than initial variability.

We adopted a consistent open-loop stimulation approach in both settings, comprising 30 s of stimulation followed by a 30-s pause, or until the heart rate returned to its baseline. This protocol was designed to balance the application of the stimulus with the heart’s natural rhythm recovery, thereby maintaining the integrity of the data.

To systematically test various combinations of stimulation parameter values (*n* = 75 for *in vivo* and *n* = 100 for *ex-vivo* experiments), we employed LHS (Stocki, 2005). LHS, a statistical method, ensured a comprehensive coverage of the parameter space, reducing the number of required experiments while maximizing the diversity and relevance of the data collected. The specific ranges of the parameters tested are detailed in Table 1. An overview of the value distributions for each stimulation parameter across various innervation conditions is available in Supplementary Material S1. This approach enabled us to precisely examine the effects of different stimulation parameters on heart function under both *in vivo* and isolated conditions.

**TABLE 1:** Summary of stimulation parameter ranges used to generate the stimulation parameter combinations for intact, bilateral rostral vagotomy, and isolated heart experiments.

	Intact^a^ (*n* = 75)	Vagotomy (*n* = 75)	Isolated (*n* = 100)
Intensity (mA)	0.2 to 1	0.2 to 2	6 to 9.7
Pulse width (μs)	50 to 200	50 to 200	25 to 1,000
Frequency (Hz)	21.3 to 42.7	10 to 40	25 to 38.5
Number of pulses	1 to 4	1 to 6	5 to 14
Delay (ms)	16 to 156.2	0 to 298.8	0 to 382

^a^ Data for intact innervation taken from Ojeda et al. (2016).

### 2.5 Data analysis

#### 2.5.1 Heart rate effect marker and stimulation charge calculation

Heart rate effects were quantitatively analyzed in vagotomized rabbits and isolated heart experiments using the methodology outlined by Ojeda et al. (2016), which involved calculating heart rate response markers for each VNS parameter combination. This approach, aimed at ensuring comparability and robustness, involved segmenting data into intervals: 30-s baselines without stimulation followed by 30 s with stimulation. This segmentation, aligned with the stimulation protocol, covered both phases for each parameter combination to ensure comprehensive data capture.

Subsequently, QRS complexes were identified in each segment of ECG data using an Energy Operator-Based Automated approach, as described by Pan and Tompkins (Pan and Tompkins, 1985). Subsequently, we calculated the heart rate modulation marker (ΔHR), representing the relative heart rate reduction for each stimulation parameter combination according to Eq. 1

$$\Delta HR=\frac{{HR}_{bl}-{HR}_{stim}}{{HR}_{bl}},$$ (1)

Where ${HR}_{bl}$ is the mean heart rate before stimulation, and ${HR}_{stim}$ is the mean heart rate during stimulation (Figure 1E). This metric quantified the impact of stimulation parameter combinations on heart rate modulation.

The physiological threshold charge, $Q_{thr}$ , was defined as the minimal charge required to reduce the heart rate by at least 5 bpm from baseline, calculated for each individual according to Eq. 2

Q=I∗PW∗NP, (2)

Where I is the stimulation intensity, PW the pulse width, and NP the number of pulses in the burst.

All stimulation parameter combinations and their corresponding relative heart rate reductions and stimulation charges were stored in a database, available in Supplementary Material S2, for further analysis.

#### 2.5.2 Statistical analysis

The statistical analysis focused on heart rate parameters and related stimulation charges across the different innervation groups. Initially, the normality of the data was assessed using the Shapiro-Wilk test. For each innervation group, the statistical analysis included calculating the mean and standard deviation for normally distributed data, and the mean and interquartile range (IQR) for data that was not normally distributed. The analyzed parameters were: baseline heart rate ( ${HR}_{bl}$ ), heart rate during stimulation ( ${HR}_{stim}$ ), maximum relative heart rate reduction ( ${\Delta HR}_{\max}$ ), the physiological threshold charge ( $Q_{thr}$ ), the charge needed to achieve maximum heart rate reduction ( $Q_{\Delta \text{HR},\max}$ ) and the respective charge normalized to the physiological threshold charge ( ${\tilde{Q}}_{\Delta \text{HR},\max}$ ).

Our statistical analysis included Kruskal-Wallis tests to assess differences between innervation groups in these parameters, supplemented by Wilcoxon Signed-Rank tests for comparing baseline heart rate with heart rate during stimulation. We also conducted a correlation analysis to explore the relationship between stimulation parameters and relative heart rate response within each innervation group, utilizing Spearman’s correlation coefficient (r).

All analyses were executed using Python scripts, which are available in Supplementary Material S2, with a significance threshold set at *p* < 0.05.

## 3 Results

### 3.1 Descriptive statistics

Stimulation parameter combinations were systematically applied across the different innervation states to assess heart rate responses, including intact innervation, bilateral vagotomy, and fully denervated condition in isolated hearts. These experiments involved a wide range of stimulation intensities, pulse widths, numbers of pulses, frequencies, and delays to evaluate their effects on heart rate. Notably, none of the parameters exhibited a normal distribution, as confirmed by the Shapiro-Wilk test (*p* < 0.001). Consequently, to accurately represent the data, all results are presented in the form of mean ± IQR. The results, shown in Table 2, indicate significant variations in heart rate response and related stimulation charges across different innervation states.

**TABLE 2:** Characteristics of stimulation experiments in different innervation states, including baseline heart rate ( ${HR}_{\text{bl}}$ ), heart rate during stimulation ( ${HR}_{\text{stim}}$ ), threshold charge ( $Q_{\text{thr}}$ ), maximum heart rate reduction ( ${\Delta \text{HR}}_{\max}$ ) with the corresponding charge ( $Q_{\Delta \text{HR},\max}$ ) and the corresponding normalized charge ( ${\tilde{Q}}_{\Delta \text{HR},\max}$ ).

	Intact^a^ (sheep, *n* = 6)	Vagotomy (rabbit, *n* = 5)	Isolated (rabbit, *n* = 4)
${HR}_{\text{bl}}$	147 ± 6 bpm	180 ± 16 bpm	155 ± 11 bpm
${HR}_{stim}$	136 ± 9 bpm*^,‡^	175 ± 15 bpm*^,‡^	128 ± 11 bpm*^,‡^
${\Delta \text{HR}}_{\max}$	40.2% ± 8.0%*	12.9% ± 6.3%*	38.5% ± 8.9%*
$Q_{\text{thr}}$	0.09 ± 0.06 μC*	0.20 ± 0.04 μC*	9.00 ± 0.75 μC*
$Q_{\Delta \text{HR},\max}$	0.63 ± 0.15 μC*	1.45 ± 0.16 μC*	54.41 ± 13.83 μC*
${\tilde{Q}}_{\Delta \text{HR},\max}$	7.7 ± 3.9	9.7 ± 3.6	6.2 ± 2.1

^a^ Intact innervation data from Ojeda et al. (2016).

*Kruskal-Wallis test for inter-group differences, *p* < 0.05. ^‡^Wilcoxon Signed-Rank test for ${HR}_{\text{bl}}$ vs. ${HR}_{\text{stim}}$ , *p* < 0.001.

In the intact innervation condition, there was a notable shift in heart rate during stimulation compared to baseline, indicating a significant chronotropic response to VNS (Table 2, Wilcoxon Signed-Rank test, *p* < 0.001). Conversely, in the bilateral vagotomy condition, the shift in heart rate during stimulation is less pronounced, showing reduced responsiveness to VNS, which however, was still statistically significant (Table 2, Wilcoxon Signed-Rank test, *p* < 0.001). Isolated hearts exhibited a more uniform and strong chronotropic response similar to the intact innervation group (Table 2, Wilcoxon Signed-Rank test, *p* < 0.001). The baseline heart rate ( ${\text{HR}}_{\text{bl}}$ ) did not exhibit a statistically significant difference among all groups (Table 2, Kruskal-Wallis, *p* = 0.0985), which was unexpected considering the comparison between heart rates from rabbits and sheep. Notably, the baseline heart rate in sheep (147 ± 6 bpm) stood out with its surprisingly high values when compared to bilateral vagotomized rabbits (180 ± 16 bpm) and isolated rabbit hearts (155 ± 11 bpm). Notably, the extent of maximum heart rate reduction ( ${\Delta \text{HR}}_{\max}$ ) was considerably different across the groups (Table 2, Kruskal-Wallis, *p* < 0.05). The bilaterally vagotomized rabbits showed a substantially diminished ${\Delta \text{HR}}_{\max}$ (12.9% ± 6.3%) when juxtaposed with the more comparable reductions observed in the innervated group (40.2% ± 8.0%) and complete denervation in isolated hearts (38.5% ± 8.9%). These results highlight the complex influence of different states of cardiac innervation on heart rate responses to VNS. The substantial differences in heart rate changes across the three conditions underscore the significant role of autonomic innervation in modulating cardiac activity in response to VNS.

Furthermore, there were significant differences in the threshold charge ( $Q_{\text{thr}}$ ) needed to elicit a physiological response among the experimental groups (Table 2, Kruskal-Wallis, *p* < 0.05). Notably, the isolated heart group required a substantially higher charge (9.00 ± 0.75 μC) compared to the other two states observed *in vivo* (0.09 ± 0.06 μC for fully innervated and 0.20 ± 0.04 μC for vagotomized conditions). This pattern of variability persisted in the charge required for maximum heart rate reduction ( $Q_{\Delta \text{HR},\max}$ ). However, upon normalizing these charges to their respective physiological thresholds ( ${\tilde{Q}}_{\Delta \text{HR},\max}$ ), the statistical disparities between the groups dissipated (Table 2, Kruskal-Wallis, *p* < 0.05), indicating that the normalized stimulus-response relationship converged across the different innervation states.

In conclusion, the significant variations in heart rate responses and related stimulation charges observed across different innervation states underscore the complex influence of cardiac innervation on the modulation of cardiac activity during VNS.

### 3.2 Correlation analysis

**FIGURE 3:**
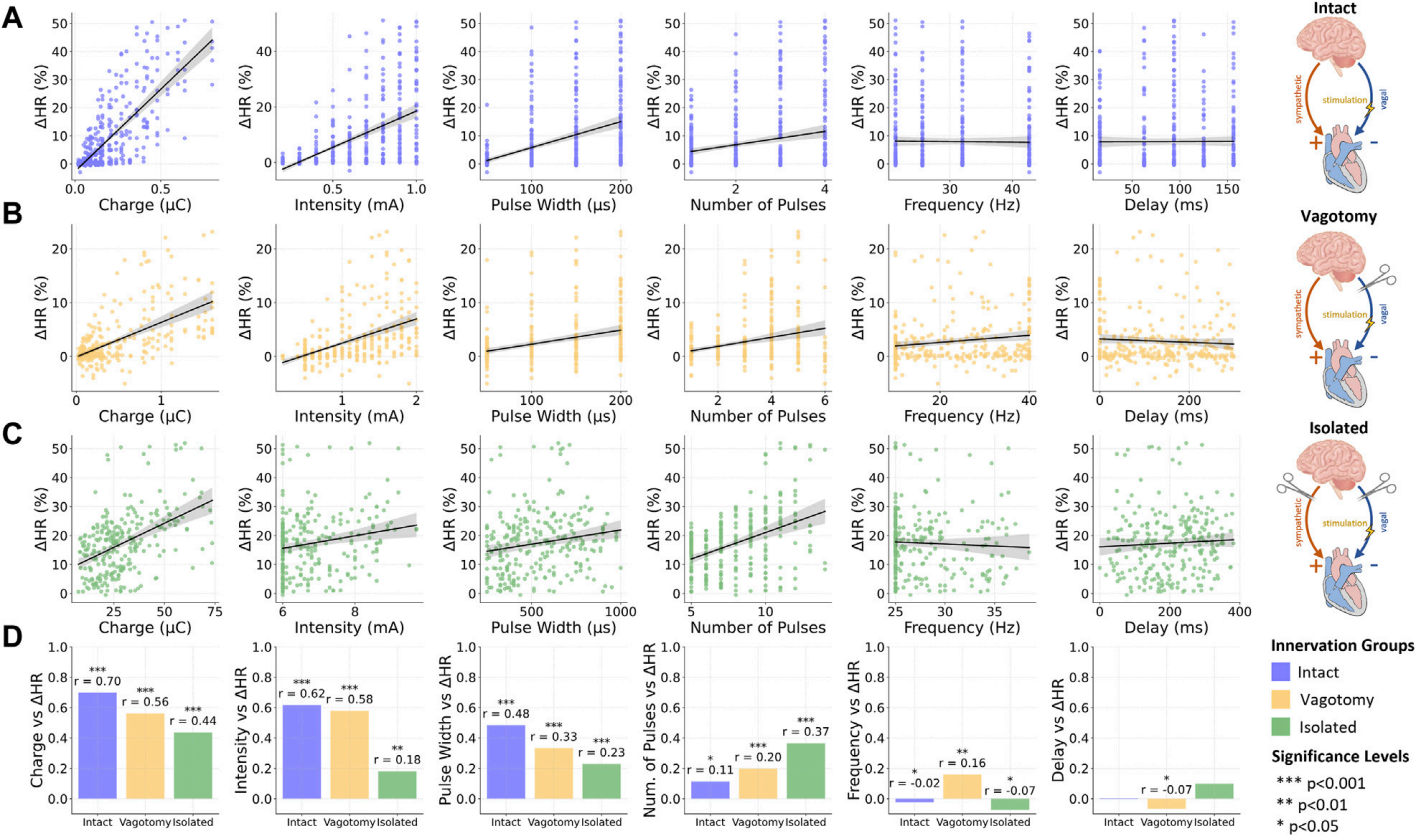
Overview of the relationship between stimulation and provoked relative heart rate reductions for **(A)** intact innervation, **(B)** bilateral rostral vagotomy, and **(C)** isolated hearts; **(D)** Summary of Spearman correlation coefficients (r) for the vagus nerve stimulation parameters and the relative heart rate reduction for different innervation groups. Data for intact innervation taken from Ojeda et al. (2016). ***p < 0.001, **p < 0.01, *p < 0.05.

In this correlation analysis, we assessed the influence of various stimulation parameters on relative heart rate changes across the three innervation groups. Figures 3A–C visually represents these relationships for each innervation group, using scatter plots to illustrate varying slopes corresponding to reported correlation coefficients. Additionally, Figure 3D summarizes the comparative impact of each stimulation parameter across different innervation states. A summary of the correlation analysis results can be additionally found in Supplementary Material S1.

#### 3.2.1 Fully innervated condition

In the fully innervated condition, moderate positive correlations were observed between heart rate reduction and both stimulation intensity (*r* = 0.62, *p* < 0.001) and pulse width (*r* = 0.48, *p* < 0.001). Notably, the charge, closely related to these two parameters along with the number of pulses, displayed a significant correlation (*r* = 0.7, *p* < 0.001), underscoring its substantial influence on heart rate modulation in fully intact innervation.

#### 3.2.2 Bilateral vagotomy condition

Transitioning to the bilateral vagotomy condition, we found that these correlations remained positive but with reduced magnitudes. Notable decreases were observed in the correlation for charge (*r* = 0.56, *p* < 0.001), intensity (*r* = 0.58, *p* < 0.001), and pulse width (*r* = 0.33, *p* < 0.001).

#### 3.2.3 Isolated heart condition

In the fully denervated condition in isolated hearts, the correlations for both intensity (*r* = 0.18, *p* < 0.01) and pulse width (*r* = 0.23, *p* < 0.001) weakened further, indicating a reduced influence on heart rate regulation. Similarly, the correlation for charge also decreased (*r* = 0.44, *p* < 0.001), reflecting the diminished responsiveness of the heart to VNS in the absence of autonomic innervation. Interestingly, the number of pulses displayed an increasing positive correlation with heart rate reduction as innervation decreased, reaching its strongest correlation in fully denervated isolated hearts (*r* = 0.37, *p* < 0.001).

#### 3.2.4 Frequency and stimulation delay

Across all innervation groups, frequency and stimulation delay exhibited very weak correlations with heart rate response, with correlation coefficients below 0.17. These findings underscore the limited role of frequency and stimulation delay in heart rate modulation through VNS within the investigated parameter value ranges.

In conclusion, the diverse correlations between stimulation parameters and heart rate changes across varying innervation states show the complex relationship between autonomic innervation and cardiac response during VNS.

## 4 Discussion

This study systematically investigated the impact of various cardiac-synchronized VNS parameters on heart rate modulation across different states of cardiac innervation. The use of cardiac-synchronized stimulation is predicated on the phase-sensitivity of the sinoatrial node to cholinergic stimulation. By timing the stimulation to coincide with specific phases of the cardiac cycle, it may be possible to achieve effective heart rate slowing with reduced stimulation intensity compared to continuous, unsynchronized VNS. This approach aims to optimize the efficacy of VNS, potentially enhancing therapeutic outcomes with lower overall stimulation levels. However, the full extent to which cardiac-synchronized VNS parameters influence heart rate responses, particularly under varying conditions of cardiac innervation, remains to be fully elucidated. Therefore, we specifically explored how these VNS parameters influence heart rate responses in scenarios of intact innervation, bilateral rostral vagotomy, and complete denervation. This is particularly relevant in clinical conditions where cardiac denervation is prevalent, such as post-heart transplantation (Awad et al., 2016), where only the efferent vagal cardiac pathways of the donor heart remain, or in diseases like diabetes mellitus (Kuehl and Stevens, 2012) or heart failure (Machado et al., 2000; Acanfora et al., 2018) that can also lead to cardiac denervation.

Previous studies have explored the feasibility of directional (Ahmed et al., 2020; Villalobos et al., 2023) and spatial-selective stimulation (Dali et al., 2018; Aristovich et al., 2021; Fitchett et al., 2021; Blanz et al., 2023; Thompson et al., 2024) and examined the influence of different stimulation parameters and stimulus shapes on the provoked cardiac effects in intact cardiac innervation (Vuckovic et al., 2008; Ojeda et al., 2016; Huffman et al., 2023). However, to this date, there has been no comprehensive examination of how main VNS parameters specifically affect cardiac responses across varying innervation scenarios. Our systematic exploration of these effects fills this crucial gap. This investigation provides insights for developing and optimizing neuromodulation strategies for the treatment of cardiac diseases tailored to the needs of patients with altered autonomic cardiac innervation.

### 4.1 Choice of stimulation parameter ranges

The selection of VNS parameter ranges was based on empirical evidence and observed physiological responses. The minimum intensities were set at physiological threshold level, while maximum levels were determined by the point where further increase did not enhance responses, reflecting a classical dose-response relationship. Our analysis focused on the linear response range to examine how stimulation parameters influenced heart rate. Other parameters, such as frequency and pulse width, were chosen within typically employed value ranges for VNS in cardiac applications. The upper limits on the number of pulses and the delay were designed to avoid continuous stimulation and ensure that the stimulation effects are confined to the intended cardiac cycle.

### 4.2 Nerve excitability and heart rate

In our study, we assessed the impact of VNS on heart rate for varying levels of cardiac innervation, including rabbits that underwent rostral vagotomy and isolated rabbit heart preparations. The results were then compared with sheep having intact innervation as reported by Ojeda et al. (2016). A lack of significant difference in baseline heart rates was observed among these groups (Table 2, Kruskal-Wallis, *p* = 0.0985). This result is surprising, considering the inherent species differences in normative heart rate ranges between rabbits (130-300 bpm) and sheep (60-120 bpm) (Milani-Nejad and Janssen, 2014), which led us to anticipate a notable discrepancy. Nevertheless, this comparison reveals two intriguing findings. First, the recorded high baseline heart rate in sheep (147 ± 6 bpm) in the study of Ojeda et al. (2016); (Kronsteiner et al., 2023) significantly exceeds the typical range reported for sheep in the existing literature (Milani-Nejad and Janssen, 2014). Second, it is noteworthy that isolated rabbit hearts exhibit a markedly lower baseline heart rate compared to those subjected to bilateral vagotomy, despite the lack of statistical significance.

This underscores the critical need to consider both the influence of medication regimens, as previously noted (Ahmed et al., 2021), and the state of cardiac innervation in the analysis of VNS effects. Furthermore, we must acknowledge the potential for an increased sympathetic outflow in the *in vivo* experiments, often a consequence of baroreflex activation due to hypotension induced by anesthetics in acute experiments, which can lead to elevated baseline heart rates. Such an elevation in heart rates could considerably affect the outcomes of a study in terms of heart rate response provoked by VNS (Paleczny et al., 2021). Therefore, careful consideration is essential when interpreting data, particularly in instances where baseline heart rates are not within the normative range reported for the respective animal model.

Despite these differences in baseline heart rate our analysis revealed that within each group, heart rates during VNS were significantly lower than their baseline values (Student’s Wilcoxon Signed-Rank test, *p* < 0.001). This observation highlights the pronounced negative chronotropic effect of VNS across all studied conditions of cardiac innervation. This finding does not only reinforce the potential of VNS as a versatile tool for cardiac therapy in various states of innervation but it also corroborates the existing literature. Specifically, our results align with the work of Kronsteiner et al. (2023), who demonstrated the feasibility of studying VNS-induced cardiac effects in *ex-vivo* isolated hearts. Furthremore this observation suggests broader applications of VNS in cardiac therapy and call for further research to explore its full potential in different clinical scenarios.

Our findings highlight a significant aspect: in vagotomized rabbits, the reduction in heart rate was relatively modest—a maximum decrease of only 12.9% ± 6.3%. This contrasts markedly with the reductions of 40.2% ± 8.0% in fully innervated condition and 38.5% ± 8.9% in fully denervated condition in isolated hearts. Several factors likely contribute to this diminished efficacy in vagotomized rabbits.

Firstly, the dampening effects of anesthetics and analgesics on cardiac responses to VNS are notable, and these effects are generally expected in *in vivo* experiments, whether in sheep or rabbits (Milani-Nejad and Janssen, 2014). This dampening is a critical factor across different animal models and experimental conditions. Secondly, in case of bilateral vagotomy, the cardiac response to VNS lacks the afferent component, which plays a crucial role in heart rate regulation. This afferent pathway is essential for the modulation of both parasympathetic and sympathetic activities via the nucleus of the solitary tract, impacting heart rate through the nucleus ambiguus and the dorsal motor nucleus of the vagus. Cutsforth-Gregory and Benarroch (Cutsforth-Gregory and Benarroch, 2017) note that integration at the level of the nucleus of the solitary tract may activate sympatho-inhibitory reflexes. Therefore, we presume that the absence of the afferent component in VNS during bilateral vagotomy leads to a lack of these sympatho-inhibitory effects, resulting in a diminished reduction in heart rate compared to conditions with intact innervation.

Moreover, a reflexive activation of sympathetic pathways can occur as the body’s response to counteract the hypotensive effects of anesthesia and analgesia. This increased sympathetic outflow might lead to reduced parasympathetic effects due to the phenomenon of “accentuated antagonism,” a cross-talk effect described by Levy (Levy, 1971). This accentuated antagonism implies an interplay between the sympathetic and parasympathetic systems, where an increase in sympathetic activity can suppress parasympathetic effects.

These considerations may explain the reduced heart rate response observed in vagotomized rabbits. Interestingly, in sheep with intact innervation, the substantial response to VNS despite the absence of the afferent component of cardiac response suggests additional factors at play. One significant aspect here is the inverse association between baseline autonomic structure and stimulation effects, as outlined by Paleczny et al. (2021). This principle posits that higher baseline heart rates are associated with greater heart rate responses to stimulation. Therefore, this inverse association may be a key factor in understanding the different responses observed between sheep and vagotomized rabbits.

In summary, these insights into the various contributing factors not only elucidate the observed differences in heart rate responses but also underscore the complexity of cardiac modulation through neural pathways. This understanding is vital for advancing clinical neuromodulation strategies and points to a promising avenue for future research aimed at optimizing therapeutic approaches.

In addition, our analysis showed significant differences in physiological threshold charges among the different innervation groups (Kruskal-Wallis, *p* < 0.05). These charges, representing the minimum charge needed to achieve at least a 5-bpm reduction in heart rate, were similar in both innervated (0.09 ± 0.06 μC) and vagotomized (0.20 ± 0.04 μC) animals. However, they were notably higher in isolated hearts (9.00 ± 0.75 μC). The increased threshold observed in isolated hearts is likely due to the reduced excitability of the nerve in an *ex-vivo* setting, as the nerve is severed from its natural physiological environment. This finding is crucial as it implies the impact of the nerve’s physiological condition on its responsiveness to electrical stimulation.

To address the variations in nerve excitability and differences in the electrode-nerve interface, such as contact quality and electrode type, we employed a normalization process. Specifically, we adjusted the charges required to achieve the maximum heart rate reduction according to the individual physiological threshold of each subject. This normalization led to comparable values across the groups: 7.7 ± 3.9 for fully innervated, 9.7 ± 3.6 for vagotomized, and 6.2 ± 2.1 for fully denervated condition in isolated hearts. Our statistical analysis (Kruskal-Wallis, *p* < 0.05) found no significant differences between these normalized values, indicating a consistent effect of VNS across different innervation states. This in turn, shows the effectiveness of normalization as a crucial step in cardiac neuromodulation research, ensuring more accurate and comparable outcomes despite inherent differences in experimental conditions.

### 4.3 Influence of stimulation parameters in different innervation conditions

Furthermore, we explored the influence of various stimulation parameters on heart rate reduction across different cardiac innervation states. Our analysis, using Spearman’s correlation coefficients, revealed a notable trend: as the level of denervation increased, the impact of the charge on heart rate reduction substantially decreased. This trend was also observed for intensity and pulse width, aligning with expectations since these parameters are intrinsically linked to charge. Specifically, in fully innervated hearts, the correlation between charge and heart rate reduction was strong, indicated by a correlation of 0.76 (*p* < 0.001). This correlation decreased to 0.56 (*p* < 0.001) in bilaterally vagotomized hearts and further declined to 0.44 (*p* < 0.001) in fully denervated condition in isolated hearts. A similar diminishing trend was observed for intensity and pulse width, suggesting that as the heart becomes more denervated, the influence of these stimulation parameters decreases.

An interesting discovery in our study was the correlation between the number of pulses and heart rate reduction across different levels of cardiac denervation. Surprisingly, we observed that this correlation increased with the level of denervation. In fully intact innervation conditions, the correlation was modest (*r* = 0.11, *p* < 0.05), but it slightly elevated in vagotomized animals (*r* = 0.2, *p* < 0.001) and reached its highest value in the completely denervated condition in isolated hearts (*r* = 0.37, *p* < 0.001). This intriguing observation may suggest the existence of compensatory mechanisms within the heart’s intracardiac processes in response to the loss of afferent input. As denervation progresses, the heart might become more reliant on the direct effects of the number of VNS pulses to modulate its rate, possibly due to the diminishing influence of neural feedback mechanisms. Furthermore, it's important to consider the experimental design, particularly in the *ex-vivo* experiments where a broader range and higher values for the number of pulses were explored. Notably, the range and magnitude of the number of pulses were comparable between the fully intact and vagotomized conditions, highlighting the specificity of this trend.

Furthermore, within the investigated parameter value ranges, we observed that frequency and stimulation onset delay exhibited weak correlations with heart rate reduction across all experimental groups. This suggests that a focus on optimizing parameters like frequency and delay may not lead to significant improvements in heart rate modulation, particularly when compared to the influence of other more impactful parameters. While frequency and delay remain essential components within the spectrum of VNS parameters, their role appears to be of secondary importance in shaping heart rate outcomes, independent of the state of innervation. This insight may guide researchers towards a more refined approach to VNS, emphasizing the investigation of parameters that have a more substantial impact on cardiac function.

In summary, our exploration of VNS parameters in varying innervation conditions reveals dynamic trends in their influence on heart rate modulation. Charge, intensity, and pulse width demonstrate diminishing effects with increased denervation, highlighting the need for parameter customization. The unexpected correlation between the number of pulses and heart rate reduction in denervated condition in isolated hearts suggests compensatory mechanisms. Conversely, frequency and delay exhibit weak correlations, emphasizing their secondary role in heart rate modulation. These findings offer valuable insights for refining cardiac neuromodulation strategies.

### 4.4 Limitations

While our study offers valuable insights into VNS effects on heart rate modulation, it is important to acknowledge its limitations. We conducted a relatively small number of experiments, but the robust statistical significance (*p* < 0.05) of our findings mitigates concerns related to sample size. Future studies with larger cohorts could further validate these results.

We did not include compound nerve action potential measurements, focusing instead on physiological outcomes to directly assess heart rate modulation by VNS. Future research incorporating compound nerve action potential measurements could provide a more comprehensive understanding of the role of afferent and efferent vagal nerve fiber populations in the mediation of cardiac responses to VNS.

The use of different electrode types and variations in nerve excitability between *in vivo* and *ex-vivo* conditions might have impacted the charges needed to elicit heart rate responses. Despite these variations, the consistency of our results across different electrode types and experimental conditions is noteworthy. This consistency underscores that our findings are not overly dependent on these specific factors, lending strength to their applicability. Furthermore, the correlation analysis for each innervation condition was self-contained and coherent, making it meaningful and viable to perform comparisons between the different influences of stimulation parameters on the provoked heart rate response across different innervation conditions and species. Additionally, to address the variations in parameter ranges in isolated heart experiments and the differing vagus nerve diameters between rabbits and sheep, we employed normalization of charges to physiological thresholds. To further enhance the robustness and validity of our findings, standardizing electrode types in future studies could be beneficial.

Lastly, our study was limited to acute heart rate effects, offering a foundation for future research to explore additional aspects of cardiopulmonary function, such as cardiac contractility, atrioventricular conduction, arterial blood pressure and respiration rate, expanding the applications of VNS in cardiopulmonary care.

## 5 Conclusion

Our findings reveal for the first time that cardiac-synchronized VNS parameters—including stimulation intensity, pulse width, and number of pulses—differentially impact heart rate modulation under various cardiac innervation conditions. The role of intensity and pulse width is particularly prominent in states with intact or partially intact innervation, suggesting the involvement of both afferent and efferent pathways in modulating heart rate. Notably, the impact of the number of pulses increases as denervation decreases, peaking in fully denervated conditions in isolated hearts. In contrast, frequency and delay play a relatively minor role in heart rate modulation, suggesting limited significance in the acute effects of VNS.

Overall, these findings underscore the importance of tailoring VNS strategies based on the cardiac innervation status, offering the potential for enhanced VNS therapy efficacy in various cardiac conditions. Furthermore, our study lays the groundwork for future investigations into the broader implications of personalized cardiac care, focusing on the optimization of neuromodulation strategies to benefit patients.

## Data Availability

The original contributions presented in the study are included in the article/Supplementary Material, further inquiries can be directed to the corresponding author.
