# Supplementary material for: Importance of cardiac-synchronized vagus nerve stimulation parameters on the provoked chronotropic response for different levels of cardiac innervation
Source: Front Physiol. 2024 May 21;15:1379936. doi: 10.3389/fphys.2024.1379936 (PMC11148559; doi:10.3389/fphys.2024.1379936)
Supplement: Supplementary file 1 [file DataSheet1.PDF]

Supplementary Material

Importance of cardiac-synchronized vagus nerve stimulation parameters on the provoked chronotropic response for different levels of cardiac innervation

Max Haberbusch*, Bettina Kronsteiner, Philipp Aigner, Attila Kiss, Bruno Karl Podesser, Francesco Moscato

*** Correspondence:** Corresponding Author: max.haberbusch@meduniwien.ac.at

1 Supplementary Data

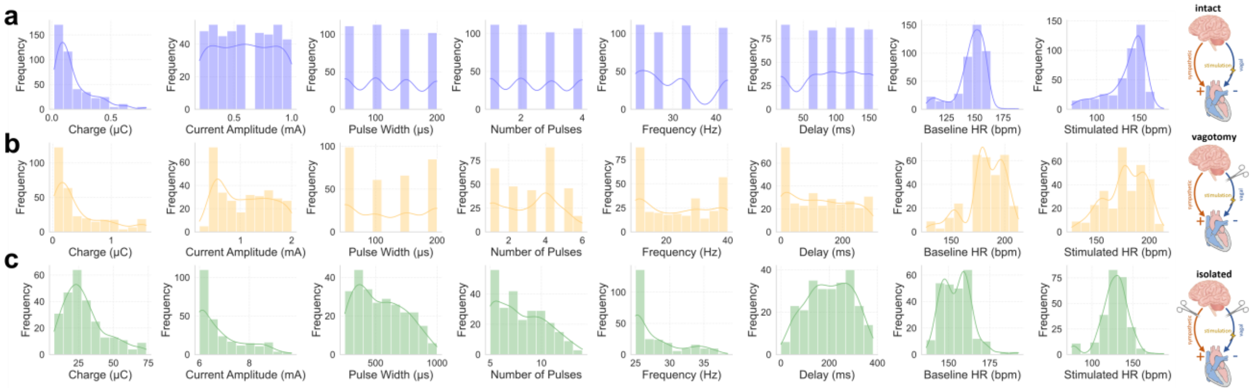

Supplementary Figure 1. Overview of the cardiac-synchronized vagus nerve stimulation parameters, baseline heart rate (HR) and heart rate during stimulation distributions for (**a**) intact innervation, (**b**) bilateral rostral vagotomy, and (**c**) isolated hearts. The data for intact innervation was taken from Ojeda et al. (2016).

**Stimulation Parameter**	**Intact** † (sheep, n=6)	**Vagotomy** (rabbit, n=5)	**Isolated** (rabbit, n=4)
Charge	0.7 ***	0.56 ***	0.44 ***
Intensity	0.62 ***	0.58 ***	0.18 **
Pulse Width	0.48 ***	0.33 ***	0.23 ***
Number of Pulses	0.11 *	0.2 ***	0.37 ***
Frequency	-0.02	0.16 **	-0.07
Delay	0.00	-0.07	0.1

Supplementary Table 1. Summary of Spearman correlation coefficients for the vagus nerve stimulation parameters and the relative heart rate reduction for the intact innervated condition,

bilateral vagotomy, and the fully denervated condition in isolated hearts. †Data for intact innervation taken from Ojeda et al. (2016). *** $p < 0.001$, ** $p < 0.01$, * $p < 0.05$.

References

Ojeda, D., Le Rolle, V., Romero-Ugalde, H. M., Gallet, C., Bonnet, J. L., Henry, C., ... & Hernández, A. I. (2016). Sensitivity analysis of vagus nerve stimulation parameters on acute cardiac autonomic responses: Chronotropic, inotropic and dromotropic effects. PLoS ONE, 11(9), e0163734.
